# Nuclear Imaging of a Pregnant Patient: Should We Perform Nuclear Medicine Procedures During Pregnancy?

**DOI:** 10.4274/Mirt.123

**Published:** 2012-04-01

**Authors:** Gonca G. Bural, Charles M. Laymon, James M. Mountz

**Affiliations:** 1 University of Pittsburgh Medical Center, Department of Radiology, Nuclear Medicine Division, Pittsburgh, PA, USA

**Keywords:** Pregnancy, nuclear medicine, diagnostic techniques and procedures, diagnostic imaging

## Abstract

Although it is extremely rare, nuclear imaging of a pregnant woman presents a unique challenge to the nuclear medicine physician because of the high concern for radiation risk to the embryo or the fetus. This challenge has been exacerbated due to recent heightened public concern of medical procedures involving radiation. This awareness also has been emphasized to the referring physicians to the extent that the risks of most nuclear medicine scans are overstressed relative to the benefit. Radionuclide procedures are reluctantly ordered by clinicians in pregnant patients, because of the malpractice fear or because of uncertainty regarding fetal radiation dose. However, when used appropriately, the benefits of nuclear imaging procedures usually outweigh the minimal risks associated with small amount of radiation even in pregnant patients.

**Conflict of interest:**None declared.

## INTRODUCTION

Radiation exposure to patients from imaging studies has been an important issue. Growing public awareness of the potential hazard of radiation has led to involvement of several organizations and the establishment of radiological protection standards, legislation, guidelines, and programs. Modern nuclear medicine imaging procedures offer significant information about disease and are often critical components of patient management. Pregnant women present a particularly difficult challenge because of the need to balance the benefit of the imaging study to the mother with the risk from the radiation exposure to the unborn embryo or fetus. 

**This Review Article is Written in Order to **

A. Provide an overview of radiation effects on the embryo or the fetus during various stages of development.

B. Relate fetal radiation doses from selected nuclear medicine procedures to the established fetal exposure limits.

C. Describe actions and precautions that could reduce radiation exposure to the unborn child.

D. Briefly discuss the current literature on nuclear imaging of a pregnant patient.

E. Describe factors entering into the cost to benefit decision analysis for nuclear medicine procedures during pregnancy and emphasize the necessity of communication of these to the referring physicians and the patient.

**A. Radiation Effects on the Embryo or the FetusDuring Various Stages of Development**

The possible effects associated with prenatal radiation exposure include immediate effects (such as fetal death or malformations) or increased risk for cancer later in life. 

**Early Gestation / First Trimester:** At this stage, the rate of fetal growth is very rapid and the fetus is at its most radiation-sensitive stage. It is believed that radiation injury during early gestation is an "all-or-nothing" effect (1). 

**Second Trimester:** The overall growth rate of the fetus has slowed during this period. However, the major organ systems are beginning to differentiate. The fetus is in its most sensitive stage to radiation. The incidence of gross congenital malformations and mental retardation are dose-related and appear to have thresholds (1).

**Third Trimester:** Irradiation during this period may deplete cell populations at very high doses (over 50 rem), but will not result in gross organ malformations (1).Before about 2 weeks gestation (the time after conception), the health effect of concern from an exposure of > 0.1 gray (Gy) or 10 rads is the death of the embryo* (2,3). If the embryo survives, radiation-induced noncancer health effects are unlikely, no matter what the radiation dose (2,3). From about 16 weeks’ gestation to birth, radiation-induced noncancer health effects are unlikely below about 0.50 Gy (50 rads) (2). Radiation may significantly effect brain development among persons exposed at 8-15 weeks’ gestation. Atomic bomb survivor data indicate that, the average IQ loss is approximately 25-31 points per Gy (per 100 rads) above 0.1 Gy (10 rads), and the risk for severe mental retardation is approximately 40% per Gy (per 100 rads), above 0.1 Gy (10 rads). The central nervous system is less sensitive in the 16- to 25-week stage of gestation. In the 16- to 25-week stage the average IQ loss is approximately 13-21 points per Gy (per 100 rads) at doses above 0.7 Gy (70 rads) (4,5).

**B. Fetal Radiation Doses From Selected NuclearMedicine Procedures and the Established Fetal Exposure Limits**

Fetal radiation doses for selected nuclear medicine procedures are given in Table 1 (6). These values can be compared with the dose to the conceptus from naturally occurring background radiation, which is approximately 0.5-1 mSv (50-100 millirem; 0.0005-0.001 Gy) for the entire period of gestation (7).Most researchers agree that a dose of <0.05 Gy (5 rads) represents non-measurable noncancer risk to the embryo or fetus at any stage of gestation. Beyond about 26 weeks, the fetus is less sensitive to the noncancer health effects of radiation exposure than in any other stage of gestation. At doses above 1 Gy (100 rads) the risks for miscarriage and neonatal death increases.International Commission on Radiological Protection Publication (ICRP) 84 “Pregnancy and Medical Radiation”, reported that “Prenatal doses from most properly done diagnostic procedures present no measurably increased risk of prenatal death, malformation, or impairment of mental development over the background incidence of these entities” (8). The commission also stated, “Fetal doses below 100 mGy (10 rad) should not be considered a reason for terminating a pregnancy (8)”.ICRP Publication 84 states that radiation induced malformations have a threshold of 50 to 100 mGy (5-10 rad). At fetal doses in excess of 500 mGy (50 rad), there can be significant fetal damage, the magnitude and type of which is a function of dose and stage of the pregnancy. A fetal dose of 100 mGy (10 rad) has a very small risk of radiation-induced cancer. At this level, there is over a 99 percent (99%) chance the exposed fetus will not develop childhood cancer or leukemia.Most standard nuclear medicine procedures produce fetal radiation doses below 50 mSv (5,000 millirem). The National Council on Radiation Protection and Measurements and the American College of Obstetricians and Gynecologists agree that the potential health risks to a fetus are not increased from most standard medical tests with a radiation dose below 50 mSv (9). Potential health risks may increase for combinations of several tests that result in radiation doses that exceed 50 mSv, depending on the dose and on the stage of pregnancy (9).

**C. Actions and Precautions that Could Reduce Radiation Exposure to the Unborn Child**

In females of child-bearing age, preprocedural questioning and testing should be made to determine who is, or could be, pregnant, prior to radiation exposure. A missed period in a regularly menstruating woman should be considered due to pregnancy, until proven otherwise.For most, but not all diagnostic imaging studies, fetal dose (and dose in general) can be reduced by a reduction in administered activity (e.g. 1/2 a typical administration) with a corresponding increase an imaging time (e.g. double) to preserve image quality.Due to the fact that the some tracers are metabolized and excreted by the kidneys, and thus accumulate in the bladder, an effective method for reducing absorbed radiation dose to the fetus is to catheterize the bladder with simultaneous provision of intravenous hydration during the procedure. Nuclear medicine physicians should be aware that some radionuclides cross the placenta and pose increased fetal risks. An important example is I-131. The fetal thyroid accumulates iodine after about 10 weeks of gestational age. High fetal thyroid doses from radioiodine can result in permanent hypothyroidism. If pregnancy is discovered within 12 h of radio-iodine administration, prompt oral administration of stable potassium iodine (60-130 mg) to the mother can reduce fetal thyroid dose. This may need to be repeated several times (8). 

**D. Current Literature on Nuclear Imaging of Pregnant Patient**

**Figure 1:**
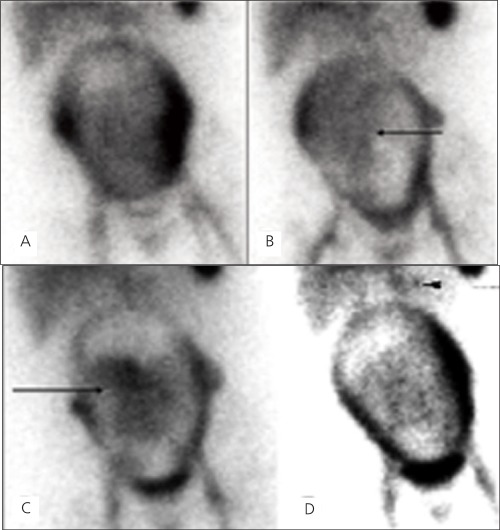
Selected images from the one minute per frame sequentialimages of an in vitro gastrointestinal bleeding scan on a 6-month-oldpregnant woman. At 4 minutes after injection (A) there is traceraccumulation in the liver, spleen, the major blood vessels and theuterus. Increased tracer accumulation is seen in lateral walls of theuterus more on the left than the right, during the beginning of thestudy. This likely represents the normal perfusion to the placenta. At15 minutes, time there is diffuse activity seen over the gestationaluterus. Faint area of mildly increased activity accumulation within theuterus is noted. This central activity changes position over the courseof the scan represents the viable fetus (B). The activity in the fetus isprobably due to radioactive break down components of Tc-99m RBCssince intact RBCs do not normally cross the placenta. At 40 minutes(C) there is seen to be more prominent activity accumulation in theviable fetus. The final image at 60 minutes (D) shows a more clearlydefined “blush” of activity in the left upper quadrant questionablefor a GI bleeding (arrowhead) and an angiography showed a gastrosplenicarteriovenous malformation (AVM)

Although it is extremely rare, a few nuclear medicine imaging procedures have been performed and reported on pregnant patients including a recent study with 18F FDG PET/CT. Six 18F-FDG PET studies were performed on five pregnant women with malignancies. The fetal radiation doses were assessed and found that the doses were significantly below the threshold for deterministic effects of radiation exposure to the fetus, and all patients ultimately delivered healthy infants without visible abnormalities (10).Bagga reported an 18 F-FDG PET/CT scan that was performed on a pregnant subject where Hodgkin lymphoma was diagnosed (11). The patient was counseled about radiation exposure and elected to have a therapeutic abortion than underwent chemotherapy after the scan. After imaging a pregnant patient with a Tc-99m macroaggregated albumin (MAA) perfusion study and a Tc-99m DTPA aerosol ventilation study, Marcus et al. have concluded that the risk to mother and fetus from an undiagnosed pulmonary embolism outweighed the small risk to the fetus from radiation (12). This conclusion was shared by Ponto (13) who performed fetal dosimetry calculations related to the studies described by Marcus et al.Baker et al. presented cases in which pregnant breast cancer patients were evaluated for metastatic disease using Tc-99m MDP bone scans (14). To minimize fetal absorbed dose, tracer injection was reduced by half and imaging time was doubled.Palestro et al. published the incidental diagnosis of pregnancy on Tc-99m MDP and Ga-67 citrate scans of a pregnant patient who was under evaluation for low back pain and discussed the estimated fetal radiation doses from these scans (15).Padit-Taskar et al. performed a study to assess the amount of radiation exposure to the fetus during breast surgery involving sentinel lymph node (SLN) mapping using technetium-99m sulfur colloid (TSC) and compared this exposure to the federal regulatory limits (16). It was concluded that the maximal fetal doses during breast surgery involving TSC for SLN mapping are small and well below levels associated with risk concerns. At the administered activity levels (0.1 mCi and 0.5 mCi), pregnancy was not be considered a contraindication for SLN mapping in breast cancer utilizing TSC.After obtaining informed consent, a gastrointestinal bleeding study was performed in a pregnant patient during the 6 months of pregnancy who presented with hematemesis and dark blood in stool at our institution (Figure 1). An example of the informed consent document is provided. Bleeding scan showed faint tracer accumulation in the left upper quadrant questionable for a GI bleed which prompted an angiography. The angiogram showed a gastrosplenic arteriovenous malformation (AVM), and the patient therefore underwent interventional embolization of the AVM. After her condition stabilized, she was discharged from the hospital. The radiation dose to the fetus for a Tc-99m RBC-in vitro scan with an administered dose of 20 mCi, was calculated as 2.52 mGy (252 millirads) at 6 months and reported (17).

**E. Factors Entering Into the Cost to Benefit Decision Analysis for Nuclear Medicine Procedures During Pregnancy and Communication of these to the Referring Physicians and the Patient**

In the rare case where a nuclear imaging study is ordered on a pregnant woman, the fetal radiation dose should be estimated by the nuclear medicine physician to provide more detailed approximation of possible risks to the fetus. Consultation with hospital medical physicists or a health physicist should be considered.The benefits and risks of a nuclear medicine procedure should be carefully discussed among the nuclear medicine physician, the referring physician, and most importantly the pregnant patient, who could be frightened.An important role of the nuclear medicine physician is to engage in a calm discussion and provide the patient the information needed to make rational decisions. This should include reassurance that the risk to the fetus is extremely small. This needs to be facilitated by outlining the potential absorbed dose to the fetus in the context of accepted standards and guidelines. The nuclear medicine physician should also emphasize that when used appropriately, the benefits of nuclear imaging procedures usually outweigh the minimal risks associated with small amount of radiation, even in pregnancy. 

*Both gray (Gy) and rad are units of absorbed dose and reflect theamount of energy deposited into a mass of tissue (1 Gy=100 rads).

## Figures and Tables

**Table 1:**
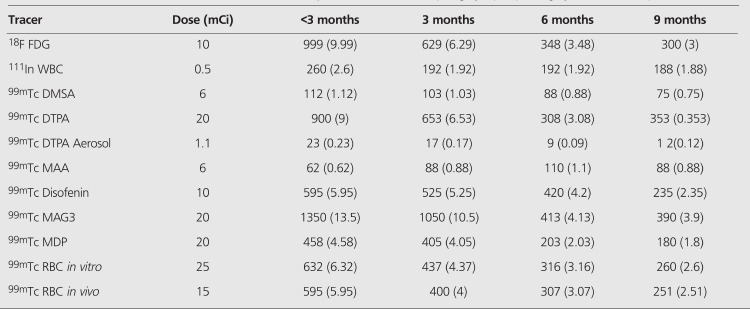
Fetal radiation doses for selected nuclear medicine procedures in milliards (milligray**) **(1 milligray = 1 milliSievert)
